# Expanding the toolbox for cryopreservation of marine and freshwater diatoms

**DOI:** 10.1038/s41598-018-22460-0

**Published:** 2018-03-09

**Authors:** Willem Stock, Eveline Pinseel, Sam De Decker, Josefin Sefbom, Lander Blommaert, Olga Chepurnova, Koen Sabbe, Wim Vyverman

**Affiliations:** 1 Laboratory of Protistology and Aquatic Ecology, Ghent University, Krijgslaan 281-S8, B-9000 Ghent, Belgium; 2 Department of Bryophyta and Thallophyta, Botanic Garden Meise, Nieuwelaan 38, B-1860 Meise, Belgium; 3 Ecosystem Management Research Group (ECOBE), University of Antwerp, Universiteitsplein 1, B-2610 Wilrijk, Belgium; 4 Present Address: Department of Marine Sciences, University of Gothenburg, Box 461, 405 30 Göteborg, Sweden; 5 Present Address: Institut de Biologie Physico-Chimique (IBPC), UMR 7141, Centre National de la Recherche Scientifique (CNRS), Université Pierre et Marie Curie, 13 Rue Pierre et Marie Curie, F-75005 Paris, France

## Abstract

Diatoms constitute the most diverse group of microalgae and have long been recognised for their large biotechnological potential. In the wake of growing research interest in new model species and development of commercial applications, there is a pressing need for long-term preservation of diatom strains. While cryopreservation using dimethylsulfoxide (DMSO) as a cryoprotective agent is the preferred method for long-term strain preservation, many diatom species cannot be successfully cryopreserved using DMSO. Therefore, in this study, we studied cryopreservation success in six different diatom species, representing the major morphological and ecological diatom groups, using a range of DMSO concentrations and Plant Vitrification Solution 2 (PVS2) as an alternative cryoprotectant to DMSO. In addition, we tested whether suppressing bacterial growth by antibiotics accelerates the post-thaw recovery process. Our results show that the effects of cryoprotectant choice, its concentration and the addition of antibiotics are highly species specific. In addition, we showed that PVS2 and antibiotics are useful agents to optimize cryopreservation of algae that cannot survive the traditional cryopreservation protocol using DMSO. We conclude that a species-specific approach will remain necessary to develop protocols for diatom cryopreservation and to increase their representation in public culture collections.

## Introduction

Among microalgae, diatoms (Bacillariophyta) constitute the most species-rich group on Earth^1,2^. They are ecologically widespread, occurring in marine, freshwater and (semi)terrestrial habitats worldwide, and significantly contribute to primary production and to the global cycling of both carbon and nutrients^1,3^. In recent years, diatoms are increasingly being recognised for their large potential in biotechnological applications, as they produce high-value compounds^4–6^ which can be used in medicine and as food supplements. Likewise, many diatoms are rich in lipid content, making them promising candidates for biofuel production^7^. Their porous silica based cell wall, one of the main characteristic features of diatoms, has many potential nanotechnology applications, including targeted drug delivery^8,9^. Diatoms exhibit pronounced intraspecific variation in e.g. optimum growth conditions^10,11^ or viral susceptibility^12^. The fact that genetic transformation protocols are available for an increasing number of species^13–17^ allows generating strains with useful features for research. Altogether, this results in an increasing need for long-term storage of strains with high scientific or commercial interest.

Long-term preservation of micro-algae in culture has several drawbacks. First, micro-algae evolve rapidly, resulting in genomic changes during long-term maintenance in culture^18,19^. Secondly, the peculiar diatom life cycle, during which consecutive mitotic divisions lead to a gradual cell size reduction in most diatom species^20^, leads to changes in physiology^21^ and ultimately in loss of diatom culture viability^22^. It is therefore essential that long-term preservation is achieved in a culture-independent manner, either through cryopreservation or by inducing resting stages. The latter is however not always possible as many diatom species lack a persistent dormant stage. Moreover, when resting stages can be induced, their viability generally decreases with time^23^. Cryopreservation therefore currently represents the preferred method for long-term diatom preservation, as it also ensures genotypic stability^24^.

Over the past years, many different protocols and cryoprotective additives (CPAs) have been tested in order to increase the post-thaw survival rate of microalgae^25–28^. As yet, there is no universal protocol for the cryopreservation of diatoms, which is not surprising considering the substantial heterogeneity (e.g. morphology, physiology, ecology) within this group. Freshwater diatoms, for instance, have proven particularly difficult to cryopreserve^29^. In view of the growing interest in diatoms, both within fundamental and applied research, there is a pressing need to improve existing cryopreservation protocols^30–32^.

Today, dimethylsulfoxide (DMSO) is the preferred CPA for algae, including diatoms^25^, as it dehydrates the cell during cryopreservation, thus preventing the formation of lethal ice crystals in the intracellular environment. Plant vitrification solution 2 (PVS2) is the main cryoprotectant used for plants. Besides dehydrating the cells, it also alters the thermal properties of intracellular water, thereby promoting “vitrification” (i.e. the formation of *‘glass’*) in the aqueous domain of the cells, which prevents the formation of ice crystals^33^. Vitrification has been suggested as a potential cryopreservation strategy for microalgae^34^ and has recently been proven successful for cryopreservation of two marine diatom species (*Nitzschia closterium* f. *minutissima* and *Chaetoceros muelleri*), in combination with encapsulation of these diatoms in alginate beads^35,36^. However, encapsulation in alginate beads is laborious, and hence impractical for high throughput cryopreservation. Moreover, the high concentration of sucrose in PVS2 promotes bacterial growth, which may interfere with post-thaw diatom recovery. Potentially, applying antibiotics during the thawing process could solve this issue but has, to our knowledge, not been tested yet.

**Figure 1:**
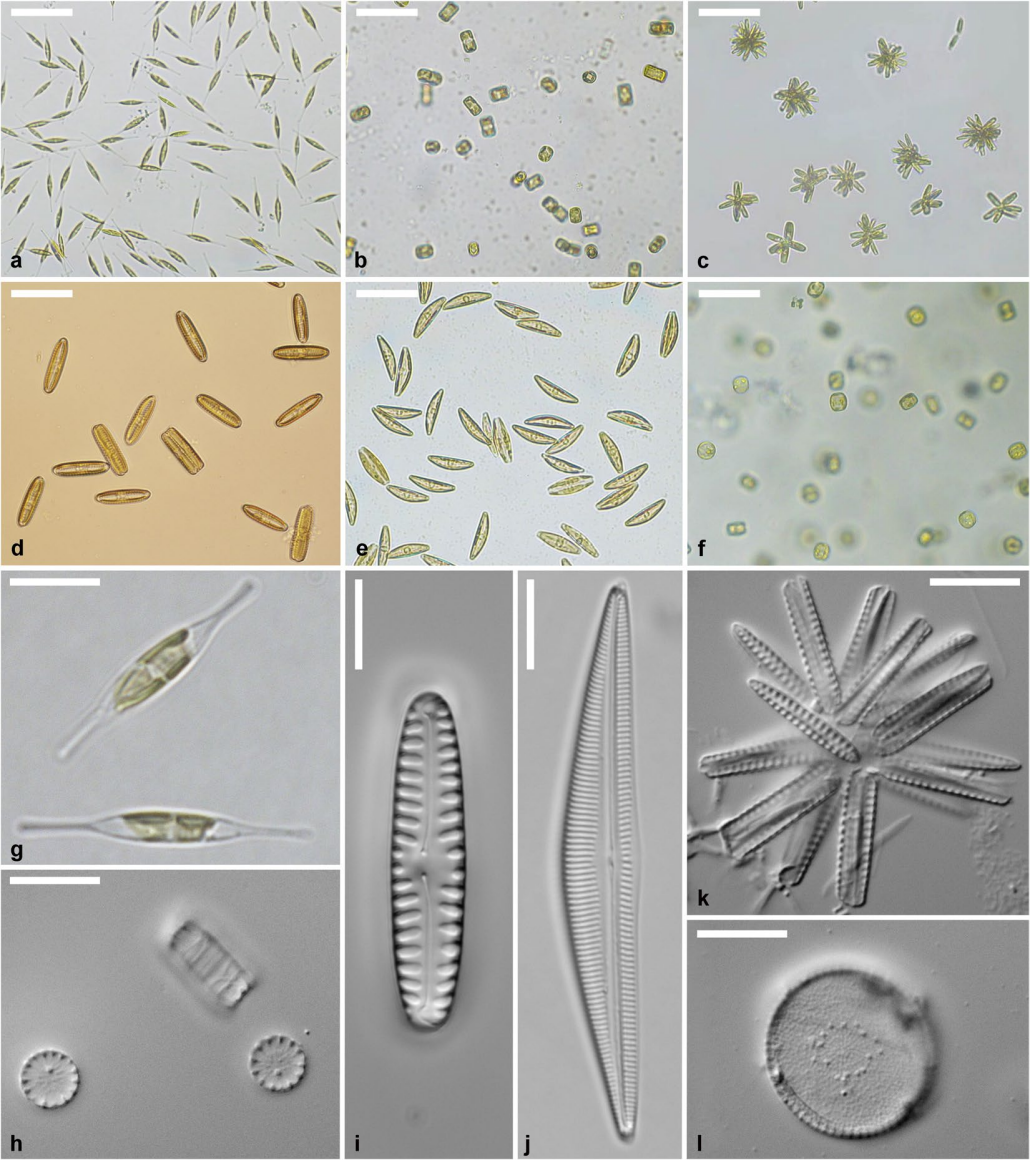
Images showing the diatom strains used for the experiments. **(a–f)** Images of living cultures of (a) *C. closterium* strain DCG 0623, (b) *C. meneghiniana* strain DCG 0273, (c) *O. guenter-grassii* strain DCG 0448, (d) *P. borealis* strain DCG 0662, (e) *S. robusta* strain DCG 0105, and (f) *T. weissflogii* strain DCG 0320. Scale bar = 50 µm. **(g)** Image of living cells of *C. closterium* strain DCG 0623. Scale bar = 10 µm. **(h–l)** Images of oxidized culture material of (h) *C. meneghiniana* strain DCG 0273, (i) *P. borealis* strain DCG 0662, (j) *S. robusta* strain DCG 0105, (k) *O. guenter-grassii* strain DCG 0448, and (l) *T. weissflogii* strain DCG 0320. Scale bar = 10 µm.

In this study, we tested whether (i) vitrification using PVS2 without prior encapsulation in alginate beads is an alternative to DMSO for diatom cryopreservation, (ii) cryopreservation success using DMSO and PVS2 can be improved by changing CPA concentrations, and (iii) addition of antibiotics in the culture medium used for post-thaw cultivation improves post-thaw recovery success. We successfully cryopreserved six diatom species (*Cylindrotheca closterium, Cyclotella meneghiniana, Opephora guenter-grassi, Pinnularia borealis, Seminavis robusta* and *Thalassiosira weissflogii*) representing the main morphological (pennate araphid, pennate raphid and centric) and ecological (freshwater, terrestrial and marine; planktonic and benthic) diatom groups (Fig. 1, Table 1).

**Table 1:** Species characteristics.

Species	BCCM/DCG accession	Habitat	Morphology	Standard culturing conditions
*Cylindrotheca closterium*	DCG 0623	Marine, benthic	Pennate, raphid	Guillard’s F/2, 18 °C, 12:12 h, 50 µmol m^−2^. s^−1^
*Cyclotella meneghiniana*	DCG 0273	Freshwater, planktonic	Centric	WC, 18 °C, 12:12 h, 50 µmol m^−2^. s^−1^
*Opephora guenter-grassii*	DCG 0448	Marine, benthic	Pennate, araphid	Guillard’s F/2, 18 °C, 12:12 h, 50 µmol m^−2^. s^−1^
*Pinnularia borealis*	DCG 0662	Terrestrial	Pennate, raphid	WC, 18 °C, 12:12 h, 5–50 µmol m^−2^. s^−1^
*Seminavis robusta*	DCG 0105	Marine, benthic	Pennate, raphid	Guillard’s F/2, 18 °C, 12:12 h, 50 µmol m^−2^. s^−1^
*Thalassiosira weissflogii*	DCG 0320	Marine, planktonic	Centric	Guillard’s F/2, 18 °C, 12:12 h, 50 µmol m^−2^. s^−1^

Overview of strains used, their accession number of the BCCM/DCG culture collection, the habitat type from which the strains were isolated, their main morphological features and standard culture conditions.

## Results

### Phytotoxicity assessment of cryoprotectants

Using pulse amplitude modulated (PAM) chlorophyll fluorimetry we monitored potential changes in photosystem II (PSII) light use efficiency of cultures when exposed to CPAs. To establish a measure of phytotoxicity for each CPA, the half maximal effective concentration (EC50) was calculated, which is the concentration at which 50% of the maximal decrease in the photosystem II (PSII) light use efficiency is observed. In all species, the PSII light use efficiency decreased upon exposure of cells to both DMSO and PVS2, but the response was stronger with DMSO than for PVS2, resulting in an EC50 of 12.2–27.5% for DMSO and 16.9–93.1% v/v for PVS2, respectively (Table 2). The negative response was already evident within the first 20 minutes, after which the PSII light use efficiency stabilised and remained stable for the duration of the time measured (data not shown). Because *P. borealis* is substantially more light sensitive than the other five species (Pinseel, own observations), it was not subjected to actinic light during the phytotoxicity assessment. This is most likely why *P. borealis* displayed considerably higher EC50 values (27.5% in DMSO; 93.1% in PVS2) than all other treated species (Table 2). Based on these results we employed a conservative approach and set the higher concentration limits to 12.5% for DMSO and 20% for PVS2, respectively.

**Table 2:** Phytotoxicity of different concentrations of cryoprotectants.

species	DMSO (v/v %)		PVS2 (v/v %)	
	EC50	±SE	EC50	±SE
*C. closterium*	14.76	0.09	27.13	0.27
*C. meneghiniana*	12.58	0.31	16.87	0.28
*O. guenter-grassii*	13.51	0.27	18.73	0.29
*P. borealis*	27.53	1.06	93.10	28.14
*S. robusta*	12.19	0.17	18.39	0.81
*T. weissflogii*	15.69	0.08	30.45	0.95

The phytotoxicity is expressed as EC50 after 20 (*S. robusta, C. closterium*, *O. guenter-grassii* and *P. borealis*) or 30 (*T. weissflogii* and *C. meneghiniana*) minutes of exposure. The standard error (SE) is given.

### Post-thawing recovery

At least one cryopreservation and recovery procedure per diatom species was successful and strains were able to recover after one week storage in the vapour phase of liquid nitrogen at −172 °C to −185 °C. Successfully cryopreserved strains showed no obvious morphological aberrations (cell size, degree of silicification) after cryopreservation and recovery. No single treatment was successful for all diatoms tested and treatment effects were highly variable between species.

**Figure 2:**
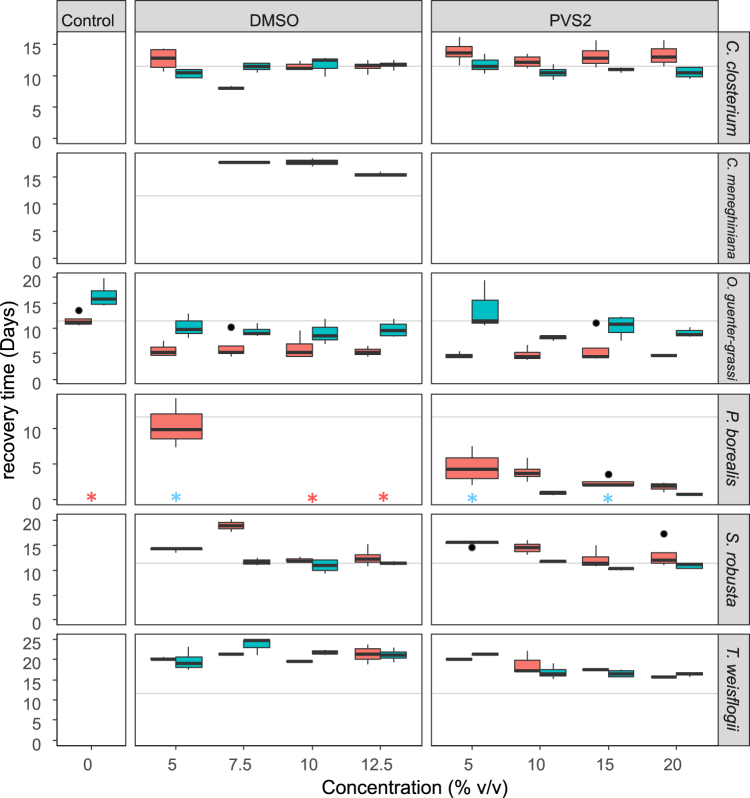
Recovery times of the tested diatoms after cryopreservation. Boxplots showing the time until the culture reaches mid exponential phase (X_0_) after cryopreservation for the different species. The different cryopreservation treatments are indicated from left to right with their respective concentrations. Recovery time without antibiotics is indicated in red, with antibiotics in blue. Treatments in which diatoms survived and recovered, but for which X_0_ could not be calculated are indicated with an asterisk.

To identify the best practice treatments we compared the time needed to reach mid-exponential phase (X_0_) (Fig. 2) and the maximum growth rate (µ_max_) during recovery (Supplementary Fig. S1). To test for general effects of each variable (i.e. species, CPA, CPA concentrations, antibiotics and their interactions) on X_0_ and µ_max_, linear models were fitted with X_0_ and µ_max_ expressed as a function of all variables. With respect to X_0_, all variables were highly significant (P < 0.0001) and were therefore included in the final model (detailed in Supplementary information: model outputs). In contrast to X_0_, the addition of antibiotics *per se* did not significantly influence µ_max_, but was instead dependent on the CPA choice and species (P < 0.0001). Both final models (X_0_ and µ_max_) were highly significant (P < 0.0001). Based on the models, there were strong interspecific differences depending on CPA choice, CPA concentration and antibiotics treatment. We therefore tested the effects separately for each diatom species.

*Cylindrotheca closterium* survived cryopreservation in all treatments, except in the control (no CPA). The recovery time of *C. closterium* differed significantly (P = 0.0013) between CPA and CPA concentrations, with a faster recovery time when preserved in DMSO compared to PVS2. However, when antibiotics were present the recovery times became shorter in PVS2 treatments and were comparable to DMSO. The addition of antibiotics did not have an effect in the DMSO treatments, and the shortest recovery time for *C. closterium* was when treated with 7.5% DMSO without antibiotics (Fig. 2).

*Cyclotella meneghiniana* displayed very poor recovery, resulting in only a few available data points. As a consequence, we did not include it in the species-specific statistical analysis and we limit the results for this species to describing the general trends for its recovery. *Cyclotella meneghiniana* was only successfully cryopreserved using DMSO as CPA and without antibiotics, however not all replicates survived. The recovery rate was highest in the 12.5% DMSO treatment without antibiotics (Fig. 2). In successful treatments, *C. meneghiniana* recovered slowly, indicating that survival during cryopreservation was very low (i.e. likely not more than one or a few cells per vial).

*Opephora guenter-grassii* survived cryopreservation in all treatments, including the controls. The recovery time, however, was significantly shorter (P < 0.0001) in the CPA treated cultures compared to the control, with no significant difference in recovery between DMSO and PVS2 treatments. Although the addition of antibiotics resulted in significantly longer recovery times (P < 0.0001) and lower maximum growth rates (P < 0.0001) (Fig. 2; Supplementary Fig. S1), *O. guenter-grassii* finally reached distinctly higher densities in the treatments with antibiotics compared to those without antibiotics (Supplementary Fig. S2).

*Pinnularia borealis* survived all treatments, including the controls (Fig. 2). Due to the characteristically slow growth of *P. borealis* in combination with low cell densities, a relatively high number of data points had to be removed from the statistical analysis, giving the false impression that there was no survival in some treatments (Fig. 2). When cryopreserved with PVS2, *P. borealis* recovered significantly faster (P < 0.0001) and displayed higher growth rates. There was a tendency toward shorter recovery times with higher concentrations of PVS2, however this was not statistically significant. Although antibiotics treatment had no significant effect on the recovery time (Fig. 2), a significantly negative effect on the maximum growth rates in all treatments (P = 0.0004) was observed (Supplementary Fig. S1). Additionally, the growth curves (Supplementary Fig. S2) illustrate that although PVS2 treated cultures in antibiotics had a slightly shorter recovery time, the final cell densities were considerably lower than those without antibiotics.

*Seminavis robusta* survived all CPA treatments, but not the controls. Similar to *C. closterium*, the presence of antibiotics resulted in significantly shorter recovery times (P < 0.0001) and significantly higher growth rates (P < 0.0001). In general, the use of higher CPA concentrations resulted in shorter recovery times and higher growth rates. Recovery time was not markedly influenced by the CPA used. With regard to X_0_, the interaction between the use of antibiotics and the CPA treatment was significant (P = 0.0083); the higher the CPA concentration, the smaller the difference in recovery time between cultures cryopreserved with and without antibiotics (Fig. 2).

*Thalassiosira weissflogii* survived cryopreservation in all treatments, but not the controls. Although *T. weissflogii* treated with antibiotics reached considerably higher cell densities (Supplementary Fig. S2), no significant effect of antibiotics was found on either the recovery time (P = 0.942) or the maximum growth rate (P = 0.0946). Cryopreservation in PVS2 resulted in shorter recovery times than cryopreservation in DMSO, with the higher PVS2 concentrations giving the best results in terms of recovery time (P < 0.0001).

## Discussion

We compared the efficiency of two different cryoprotective agents (CPAs), DMSO and PVS2, for the cryopreservation of six different diatom species, representing the main morphological and ecological groups. Moreover, we tested whether application of antibiotics in culture medium can improve post-thaw recovery. Here we report, to our knowledge for the first time, that PVS2 without the laborious procedure of encapsulation is a valuable CPA alternative to the commonly used DMSO. Furthermore, the addition of antibiotics can substantially improve post-thaw recovery in some species by preventing the diatoms being outcompeted by bacteria. Overall, we found large interspecific differences in the optimal cryopreservation method, with species demonstrating different recovery rates depending on the CPA used and its concentration, as well as the presence or absence of antibiotics. Finally, reducing the CPA concentration to a non-toxic or only mildly toxic yet still effective range can significantly improve cryopreservation success. Our results confirm that there is no ‘golden standard’ protocol for cryopreservation of diatoms, and that optimisation of the technique is still to a large extent a species-specific, empirical process^30^. However, the results of this study can help optimizing species-specific cryopreservation protocols.

Surprisingly, out of the six tested diatom species, the more commonly used CPA DMSO was more suitable for only two species (*C. closterium* and *C. meneghiniana*). *Cylindrotheca closterium* survived in PVS2, but recovered faster when treated with DMSO, whereas *C. meneghiniana* did not survive the PVS2 treatment. *Pinnularia borealis* and *T. weissflogii* recovered significantly faster using PVS2 compared to DMSO, and no significant difference between PVS2 and DMSO treated cultures was seen in *O. guenter-grassii* and *S. robusta*.

Whereas DMSO is not known to benefit bacterial growth, the sucrose-rich composition of PVS2 can be used as a nutrient source by bacteria after thawing, thereby potentially delaying or preventing recovery of a healthy diatom culture. In this study we investigated the potential benefits of using antibiotics during post-thaw recovery. We showed that two diatom species (*C. closterium* and *S. robusta*) indeed recovered faster in the presence of antibiotics in the PVS2 treatments. Moreover, in *S. robusta* the positive effect of antibiotics increased with decreasing CPA concentrations. Interestingly, this effect was observed in both DMSO and PVS2-treated cultures, suggesting that when the initial number of viable cells is low (as a consequence of low CPA concentrations), antibiotics will suppress bacterial growth long enough for the surviving cells to recover and start dividing, while in the absence of antibiotics, fast bacterial growth appears to prevent recovery of the relatively few surviving algal cells. However, the effect of the antibiotic treatment was highly species-specific. The addition of antibiotics did not have any effect on the recovery of *T. weissflogii*, while *O. guenter-grassii* recovered more slowly, *P. borealis* reached lower maximum growth rates and *C. meneghiniana* did not recover at all. It is possible that the highly variable outcomes of the antibiotics treatments were due to the composition of the antibiotics mix used in this study not being optimised for each species individually. Alternatively, some species might benefit from bacterially derived compounds^37^, thus exhibiting lower growth when bacteria are suppressed. Overall, our results show that antibiotics treatments during thawing can be beneficial for diatom culture recovery. Alternatively, to circumvent the additional stress caused by the toxicity of antibiotic agents during the post-thaw recovery, cultures could be made axenic prior to cryopreservation. Nevertheless, obtaining axenic cultures is often challenging and requires extensive and repeated antibiotics treatments. Furthermore, the cryopreservation of axenic cultures is additionally challenging as the protocol requires extensive manipulations. Adding relatively small concentrations of antibiotics to recently thawed cultures might therefore prove less labour-intensive for many culture collections.

Surprisingly, two diatom species, *O. guenter-grassii* and *P. borealis*, were able to survive cryopreservation without addition of CPAs. Tolerance to ultralow temperatures, including −180 °C, was already observed for *P. borealis* in earlier studies testing the freezing tolerance of diatoms^38,39^. Although the molecular mechanisms that allow survival of *O. guenter-grassii* and *P. borealis* cells at ca −180 °C without CPA addition remain unknown, it is noteworthy that whereas these species are not closely related, both have been isolated from relatively extreme environments. The *O. guenter-grassii* strain used has been isolated from an intertidal sand flat in the Netherlands and the *P. borealis* strain used has been retrieved from a terrestrial soil sample in Longyearbyen (Spitsbergen, High Arctic). Both of these environments are characterised by a high risk of desiccation^40^ and potentially large diurnal and seasonal temperature fluctuations^41–43^. It has previously been shown that terrestrial diatoms, including *P. borealis*, indeed show higher tolerances to desiccation and temperature extremes compared to their freshwater relatives^38–40^.

Our experiments suggest that cell characteristics which may have evolved as an adaption to environmental characteristics could play a major role in determining whether a diatom species (or strain) is able to survive ultra-low temperatures and that cryopreservation success could largely depend on the species ecology (habitat type). In this context, cell wall properties such as the amount of cell-wall associated polysaccharides or the degree of cell wall silicification could play an important role^32^. Differences in cell size might be another factor explaining intraspecific differences in cryopreservation success^44^. In order to confirm that inter- and intraspecific differences in cryopreservation success indeed originate from ecophysiological differences, more species should be tested, in particular using multiple strains of different geographical origin and different life cycle stages of the same species.

In conclusion, although our results confirm that cryopreservation procedures remain highly species-specific, we show that PVS2 (without encapsulation) is a highly feasible alternative to DMSO. In fact, the majority of the tested diatom species displayed shorter recovery times when treated with PVS2. Also, we show that antibiotics may aid post-thaw recovery in species where extensive bacterial growth is an issue. The distinct interspecific variation in recovery after cryopreservation suggests that cell damage during cryopreservation and/or the mechanisms of cell repair differ strongly between species. We found that cryopreservation success could be linked to the ecophysiology/ecology of the species. More species and more strains per species should be tested to confirm this pattern. As a consequence, no single cryopreservation protocol can be designed that is suitable for the majority of diatom species. Therefore, it will remain imperative to design and test cryopreservation protocols separately on a species-specific basis. Overall, our results confirm that cryopreservation is still largely an empirical tool since the underlying biological mechanisms of cell injury during freezing and thawing are not fully understood^45^.

## Materials and Methods

All experiments were completed within the facilities of the BCCM/DCG diatom culture collection at Ghent University (http://bccm.belspo.be/about-us/bccm-dcg). This is a certified (ISO 9001:2015) culture collection and works conform the European legislation concerning the Nagoya Protocol (http://bccm.belspo.be/services/mta). All strains used in this experiment are part of the BCCM/DCG culture collection. Prior to the experiments, all diatom cultures were maintained non-axenically in standard culture conditions (Table 1) for several weeks and were re-inoculated when reaching late exponential phase. All marine species were grown in Guillard’s F/2 medium^46^ and freshwater species in WC medium^47^ without pH adjustment or vitamin addition. Due to its high light sensitivity, *P. borealis* was grown at lower light conditions, than the other strains used in this study, following Pinseel *et al*.^48^.

Light microscopy (LM) and photography of live cultures were performed at a magnification of x400 or x1,000 (under oil immersion) using a Zeiss Axio Observer. A1 inverted microscope (Göttingen, Germany) equipped with a Nikon dsFi2 digital camera (Tokyo, Japan) or a Zeiss Axiovert 40 C inverted microscope (Göttingen, Germany) equipped with a Canon PowerShot G3 digital camera (Tokyo, Japan). Microscopy slides of oxidized material were obtained from the BCCM/DCG culture collection. Slides were obtained by harvesting 2 ml of a culture in late exponential growth phase and cleaning the silica valves by oxidation with 3 ml 69% nitric acid or 30% hydrogen peroxide at 60 °C for 2 to 6 days. The oxidized material was washed 8 times with distilled water before being mounted in Naphrax (PhycoTech, St Joseph, USA). This material predates the experiment for most strains, explaining the differences in cell size between the living and the oxidized material of some strains. Photographing of oxidized material was performed at x1,000 magnification under oil immersion using a Zeiss Axiophot 2 Universal microscope (Jena, Germany) equipped with an AxioCam MRm camera (Jena, Germany). Cultures of *C. closterium* could not be oxidized due to their fragile valves.

### Toxicity assessment of cryoprotectants

The toxicity of the two CPAs DMSO and PVS2 (30% v/v glycerol; 15% v/v ethylene glycol; 15% v/v DMSO; 0.4 M sucrose) was assessed for different concentrations (for DMSO 5, 7.5, 10, 12.5 and 15% v/v; for PVS2 5, 10, 15, 20, 25 and 30% v/v) for each of the six diatom species (Supplementary Table S1). The DMSO concentration range was chosen based on the conventionally used concentrations for diatoms^30^. The PVS2 concentrations used spanned the CPA concentrations generally used for algae^25,30^. Exponentially growing cultures were pipetted (150 µL) into black 96-well plates. CPAs (150 µL) were added to reach the final concentrations listed above. CPAs and cultures were well mixed and the phytotoxicity was assessed using a MAXI Imaging PAM fluorimeter, M-series (Walz, Effeltrich, Germany) equipped with an IMAG-K4 camera and mounted with IMAG-MAX/F filter. The toxicity was measured as the decrease in photosynthetic efficiency of Photosystem II (PSII)^49^ compared to the control (without CPA) after 20 minutes (*C. closterium, O. guenter-*grassii, *P*. *borealis* and *S. robusta*) or 30 minutes (*C. meneghiniana* and *T. weissflogii*) of exposure time. This matched the time the respective species were exposed to the CPA before being frozen (see below). All species were exposed to actinic light (15 µM photons m^−2^ s^−1^) during this period, except for *P. borealis* due to its high light sensitivity. The effect of the CPA on cell health was then reported as EC50, the CPA concentration at which 50% of the maximal toxicity effect is observed. The EC50 was calculated in R (version 3.3.3)^50^ according to Schreiber *et al*.^49^. Each treatment (CPA x concentration x species) was carried out with two technical replicates.

### Cryopreservation

Four concentrations of each CPA were selected on the basis of the results of the phytotoxicity tests: 5, 7.5, 10 and 12.5% for DMSO and 5, 10, 15 and 20% for PVS2. Cultures in late exponential growth phase (assessed microscopically and based on cell density, absence of motility, presence of actively dividing cells and cell coloration) were stored in darkness overnight, prior to concentrating them in a 50 ml falcon tube by centrifugation (1000 rcf, 5 min). The cell densities of these concentrated cultures were determined from 3 ml subsamples of each concentrated culture. For benthic and terrestrial species (*C. closterium*, *S. robusta* and *P. borealis*), 3 ml culture material was inoculated in the wells of a 12-well plate. Cells were counted on five randomly taken pictures from which the total cell density was extrapolated. Initial cell densities were ±6500 cells. ml^−1^ for *S. robusta* and ±13000 cells. ml^−1^ for *C. closterium* and *P. borealis*. For planktonic species, cell densities were determined using a Multisizer 3 coulter counter (Beckmann Coulter). Cultures contained ±8.5 × 10^5^ cells. ml^−1^ for *C. meneghiniana* and ±4 × 10^5^ cells. ml^−1^ for *T. weissflogii*. *Opephora guenter-grassii* cells clustered strongly together in culture, preventing objective cell counting by both manual counting or coulter counter.

Concentrated cultures were mixed with CPA stock solutions in 80/20 v/v in 50 ml falcon tubes (one for each CPA concentration, including the controls), resulting in equal cell densities over all treatments within each species. During the cryoprotectant incubation period, each falcon was split into four cryo vials in order to obtain four technical replicates of every treatment. The CPA incubation period equaled 20 min for *C. closterium, O. guenter-*grassii, *P*. *borealis* and *S. robusta*, and 30 min for *C. meneghiniana* and *T. weissflogii* based on prior experiments (Chepurnova, pers. comm.) where maximal post-thaw recovery was obtained using these incubation times (and using DMSO as CPA). After incubation, the vials were transferred to a pre-chilled Nalgene® Mr. Frosty freezing container (4 °C) and put at −80 °C for 1.5 h. Finally, the vials were rapidly transferred to the vapour phase of liquid nitrogen (−172 °C – −185 °C) in the cryostorage facility (Taylor-Wharton®, type 10 K). The temperature in the vapour phase of liquid nitrogen of the cryostorage facility was continuously monitored during the experiment in order to ensure that it never increased above −160 °C. All treatments were carried out in subdued light conditions (±2 µmol photons m^−2^. s^−1^).

### Post-thaw recovery

Following one week in cryostorage the cultures were thawed in a 37 °C water bath until all ice crystals had disappeared (ca. 2 min). Immediately after thawing, CPAs were diluted by adding equal aliquots of the thawed cultures into two falcon tubes containing 50 ml of their respective medium; either with or without antibiotics. For the antibiotics treatment a combination of ampicillin (bacterial cell wall synthesis inhibitor with broad spectrum activity) sodium salt, penicillin G (bacterial cell wall synthesis inhibitor effective mostly against Gram positive bacteria) sodium salt and streptomycin (bacterial protein synthesis inhibitor with broad spectrum activity) sulfate (Sigma-Aldrich, final concentration of 25 µg. mL^−1^ of each) was used. This antibiotics mix ensured that the chosen antibiotics did not (or had a minimal) impact (on) algal growth whilst the broad spectrum activity of the combined antibiotics assured sufficient growth inhibition of the bacteria present. In order to collect the suspended cells and slowly acclimatize the cells to standard culture conditions, the falcon tubes were placed in an upright position in the dark at 4 °C during 12 h. Subsequently, ca. 45 ml of the supernatant was removed, and 2 ml of the concentrated cells was transferred to 12-well plates and incubated in low light conditions (2 µmol photons. m^−2^. s^−1^) at 18 °C. Following 12 h low light incubation, cultures were placed in standard growth conditions: 23 µmol photons. m^−2^. s^−1^ at 18 °C for all species, except *P. borealis* which was kept in 5 µmol photons. m^−2^. s^−1^ at 18 °C. Recovery of the cultures was assessed by daily measurements of the minimum fluorescence values (F_0_) using pulse-amplitude-modulation (PAM) fluorimetry (Maxi imaging pam, *M-series*, Walz), starting from the second day after cryopreservation and until stationary phase was reached (Supplementary Fig. S2). Cultures were frequently checked under a Zeiss Axiovert 40 C inverted light microscope (Göttingen, Germany) to check cell condition, bacterial growth and possible contaminants.

### Statistical analysis

All statistical analyses were performed in R (version 3.3.3). A logistic function (F_0_ = L/[1 + e^ − k(X−X_0_)] with L the maximum biomass reached, k the steepness of curve and x the time) was fitted on F_0_-based growth curves from which the time needed for the culture to reach mid exponential phase (X_0_) was calculated, reflecting the recovery rate of the culture. Before fitting the function, F_0_ values, 0.95 times lower than the F_0_ value of the day before (in the decline phase) were set equal to the F_0_ of the day before to eliminate problems with fitting the function (‘negative growth’). Only significantly (p < 0.05) fitted X_0_ values from the surviving cultures were used for further processing (Supplementary Table S2). Additionally, the maximum growth rate (µ_max_) was calculated as follows: µ_max_ = ln(N_2_/N_1_)/(t_2_ − t_1_), where N_1_ = the F_0_-value at time point t_1_ and N_2_ is the F_0_-value at t_2_. This was calculated over the entire growth curve (using the sliding window approach) at three-day intervals. From these results, the maximal µ_max_ value was taken, which represents the maximum increase in biomass over a period of three days (Supplementary Table S2). This parameter is indicative for culture health. The resulting X_0_ and µ_max_ values were used as independent measures for culture fitness. To test the importance of the different parameters, linear models were fitted for X_0_ and µ_max_ separately. All parameters were modelled as factors. The different concentrations of the cryoprotectants were also considered categorical since impact of the concentrations on µ_max_ and X_0_ was not linear. The data were not transformed. The initial model included the parameters ‘diatom species’, ‘CPA treatment’, including CPA choice and concentration, and ‘antibiotics’ as well all interactions between these parameters. Model significance was tested and non-significant parameters were excluded. A final best model was selected through a stepwise algorithm using AIC as selection criterion (MASS package, 7.3–47)^51^. Next to a model over all species, a separate linear within species model was fitted for each diatom species separately, with the initial model including the parameters ‘CPA treatment’, ‘antibiotics’ and the interaction between both. Parameter significance was tested using ANOVA.

### Data availability statement

All data generated or analysed during this study are included in this published article and its supplementary information files.

## Electronic supplementary material

Supplementary information

Supplementary dataset 1

Supplementary dataset 2
