# Supplementary material for: Expanding the toolbox for cryopreservation of marine and freshwater diatoms
Source: Sci Rep. 2018 Mar 9;8:4279. doi: 10.1038/s41598-018-22460-0 (PMC5844899; doi:10.1038/s41598-018-22460-0)
Supplement: Supplementary file 1 — Supplementary information [file 41598_2018_22460_MOESM1_ESM.docx]

**Supplementary information of:**

**Expanding the toolbox for cryopreservation of marine and freshwater diatoms**

Willem Stock^1,+^, Eveline Pinseel^1,●,+^, Sam De Decker^1,+^, Josefin Sefbom^1,°,+^, Lander Blommaert^1,º^, Olga Chepurnova^1^, Koen Sabbe^1^, Wim Vyverman^1,*^

^1^ Laboratory of Protistology and Aquatic Ecology, Ghent University, Krijgslaan 281-S8, B-9000 Ghent, Belgium

^●^ Other affiliations: Department of Bryophyta and Thallophyta, Botanic Garden Meise, Nieuwelaan 38, B-1860, Meise, Belgium; Ecosystem Management Research Group (ECOBE), University of Antwerp, Universiteitsplein 1, B-2610 Wilrijk, Belgium

^°^ Current affiliation: Department of Marine Sciences, University of Gothenburg, Box 461, 405 30 Göteborg, Sweden

^º^ Current affiliation: Institut de Biologie Physico-Chimique (IBPC), UMR 7141, Centre National de la Recherche Scientifique (CNRS), Université Pierre et Marie Curie, 13 Rue Pierre et Marie Curie, F-75005 Paris, France

^+^ These authors contributed equally to this work

* author for correspondence ([wim.vyverman@ugent.be](mailto:wim.vyverman@ugent.be))

**Supplementary figures**

**Supplementary Figure S1.** **Maximal growth rate of the tested diatoms after cryopreservation.** Boxplots showing the maximal slopes of the log transformed F_0_ data. The different cryopreservation treatments are given from left to right with their respective concentrations. Treatments without antibiotics are indicated in red, with antibiotics in blue.


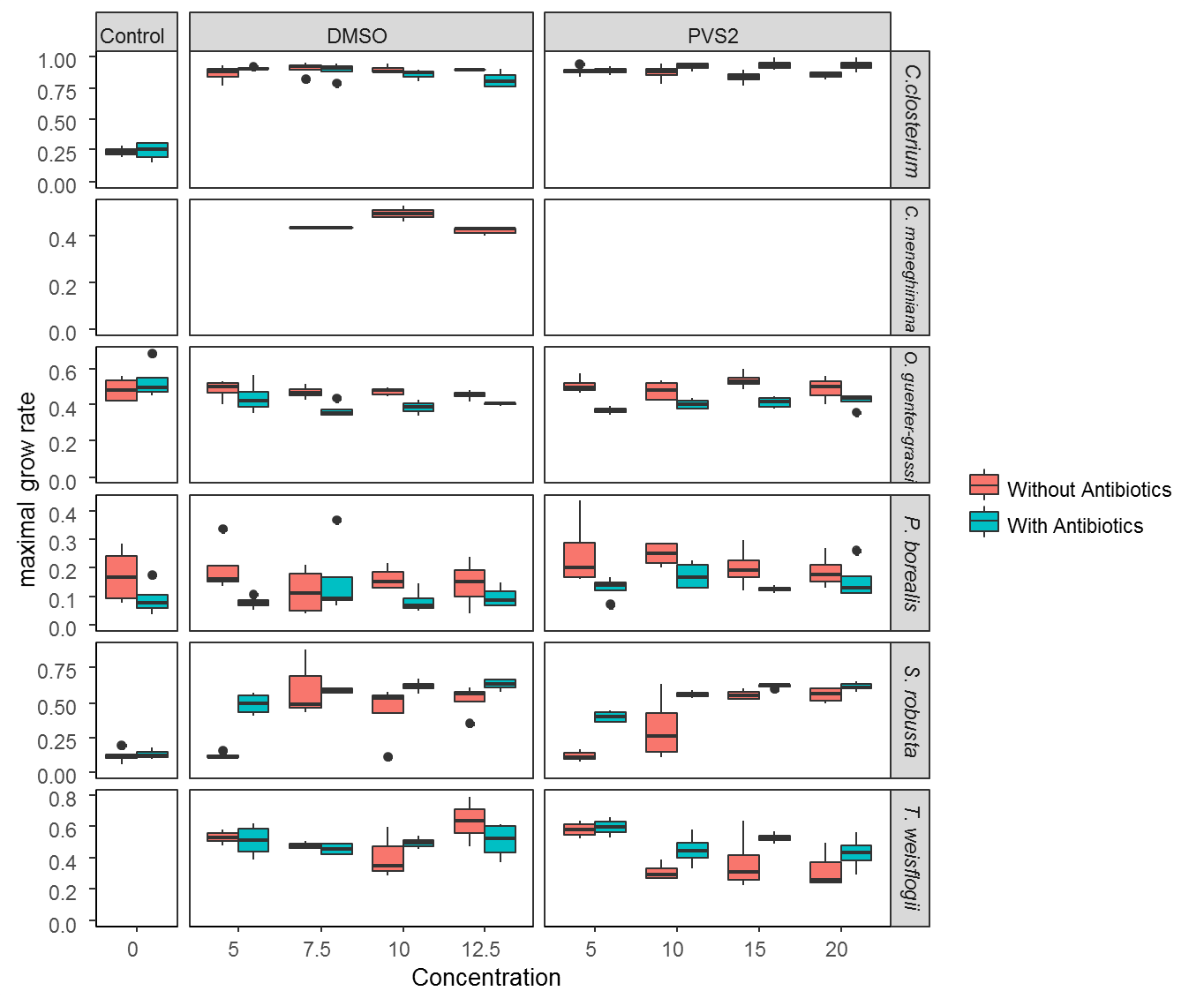


**Supplementary Figure S2. Growth curves of the tested diatoms after cryopreservation.** Figure showing the growth curves of the different species based on the daily F_0_ values. The dashed lines represent the treatments with antibiotics, the full lines without. Different colours represent the different concentrations (v/v %) of cryoprotectant used: 0 % (control) is red, 5 % is yellow; 7.5 % is green (DMSO only), 10 % is green blue, 12.5 % is blue (DMSO only), 15 % is purple (PVS2 only), 20 % is pink (PVS2 only).

**
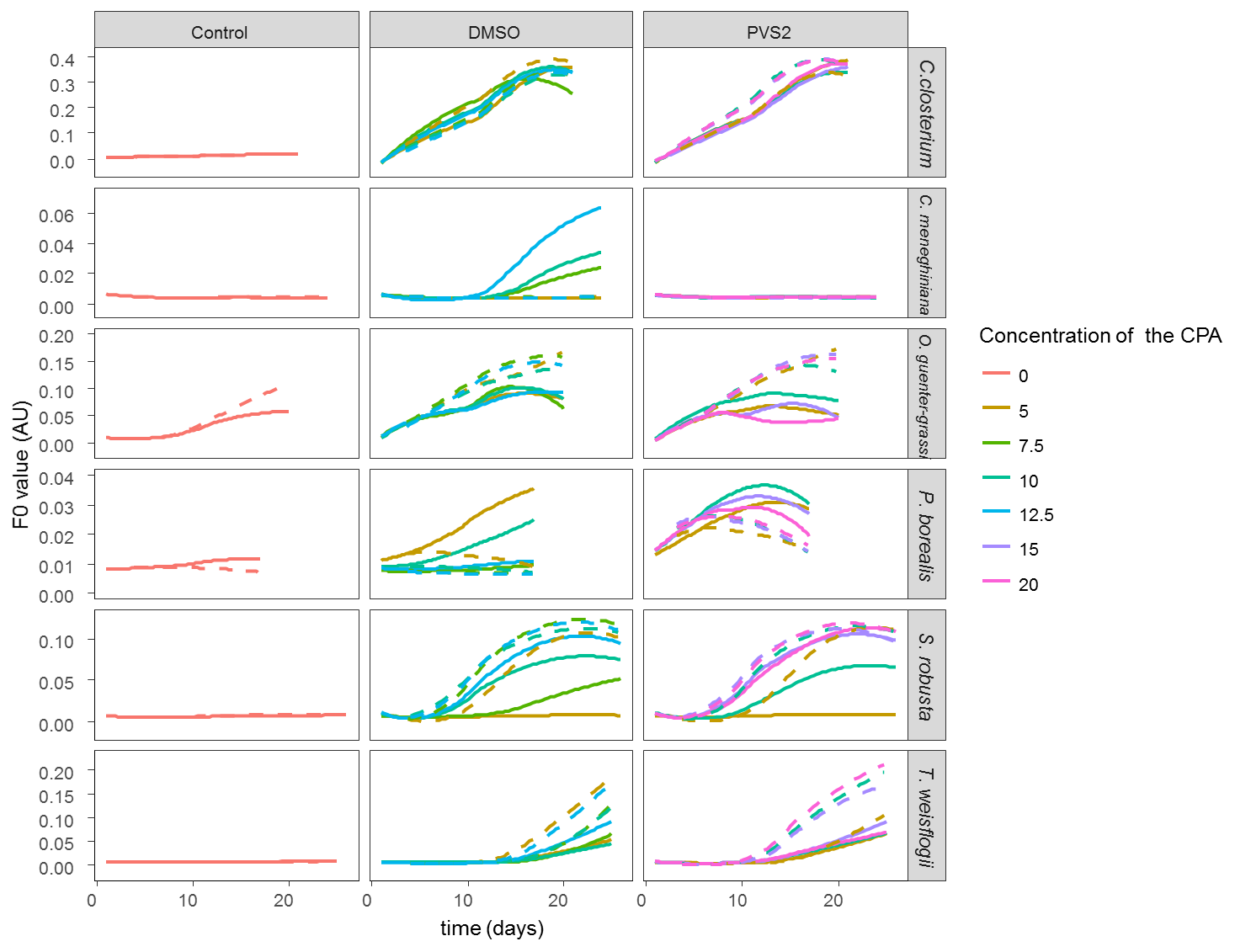
**

**Supplementary information: model outputs**

Below, all significance codes (Signif. code) refer to the following:

*** for <0.001

** for [0.001;0.01[

* for [0.01;0.05[

# **Time needed to reach mid-exponential phase (X_0_).**

*Full model:*

| Full model: X_0_ ~Diatom*Cryoprotectant*Antibiotics | | | |
| --- | --- | --- | --- |
| Adj R² | F | df | p |
| 0.896 | 30.15 | 174 | <0.0001 |

## *Significance of the terms included in the final X_0_ model:*

|  | Df | Sum of Sq | F value | P value | Signif. code |
| --- | --- | --- | --- | --- | --- |
| Antibiotics | 1 | 333 | 122.31 | <0.0001 | *** |
| Diatom | 5 | 4254 | 312.031 | <0.0001 | *** |
| Cryoprotectant | 8 | 451 | 20.659 | <0.0001 | *** |
| Antibiotics:Diatom | 4 | 414 | 37.941 | <0.0001 | *** |
| Antibiotics:Cryoprotectant | 8 | 67 | 3.049 | 0.0031 | ** |
| Diatom:Cryoprotectant | 27 | 347 | 4.712 | <0.0001 | *** |
| Antibiotics:Diatom:Cryoprotectant | 20 | 135 | 2.482 | 0.0008 | *** |

## *Models for the individual species:*

Five models (not enough data for *C. meneghiniana*) fitted only interpret p<0.01 as significant. The model per species thus becomes: X_0_ ~ Cryoprotectant + Antibiotics + Cryoprotectant:Antibiotics.

| *C. closterium* | Df | Sum of Sq | F value | P value | Signif. code |
| --- | --- | --- | --- | --- | --- |
| Cryoprotectant | 7 | 44.87 | 4.16 | 0.0013 | ** |
| Antibiotics | 1 | 13.45 | 8.73 | 0.0049 | ** |
| Cryoprotectant:Antibiotics | 7 | 61.09 | 5.67 | <0.0001 | *** |

| *O. guenter-grassii* | Df | Sum of Sq | F value | P value | Signif. code |
| --- | --- | --- | --- | --- | --- |
| Cryoprotectant | 8 | 322.6 | 9.61 | <0.0001 | *** |
| Antibiotics | 1 | 349.6 | 83.32 | <0.0001 | *** |
| Cryoprotectant:Antibiotics | 8 | 48.2 | 1.44 | 0.205 |  |

| *P. borealis* | Df | Sum of Sq | F value | P value | Signif. code |
| --- | --- | --- | --- | --- | --- |
| Cryoprotectant | 4 | 171.23 | 12.93 | <0.0001 | *** |
| Antibiotics | 1 | 14.25 | 4.31 | 0.0556 |  |
| Cryoprotectant:Antibiotics | 1 | 2.06 | 0.62 | 0.442 |  |

| *S. robusta* | Df | Sum of Sq | F value | P value | Signif. code |
| --- | --- | --- | --- | --- | --- |
| Cryoprotectant | 7 | 88.35 | 7.12 | <0.0001 | *** |
| Antibiotics | 1 | 64.34 | 36.3 | <0.0001 | *** |
| Cryoprotectant:Antibiotics | 5 | 38.47 | 4.34 | 0.0033 | ** |

| *T. weissflogii* | Df | Sum of Sq | F value | P value | Signif. code |
| --- | --- | --- | --- | --- | --- |
| Cryoprotectant | 7 | 191.77 | 8.191 | <0.0001 | *** |
| Antibiotics | 1 | 0.02 | 0.005 | 0.942 |  |
| Cryoprotectant:Antibiotics | 7 | 15.59 | 0.666 | 0.698 |  |

# **Maximal grow rate (µ_max_).**

*Full model:*

| Full model: µ_max_ ~ Diatom * Cryoprotectant *Antibiotics | | | |
| --- | --- | --- | --- |
| Adj R² | F | df | P |
| 0.9066 | 36.91 | 243 | <0.0001 |

## *Significance of the terms included in the final µ_max_ model:*

|  | Df | Sum of Sq | F value | P value | Signif. Code |
| --- | --- | --- | --- | --- | --- |
| Diatom | 5 | 15.597 | 485.53 | <0.0001 | *** |
| Cryoprotectant | 8 | 1.473 | 28.655 | <0.0001 | *** |
| Antibiotics | 1 | 0.013 | 2.06 | 0.1525 |  |
| Diatom:Cryoprotectant | 33 | 3.231 | 15.241 | <0.0001 | *** |
| Diatom:Antibiotics | 4 | 0.533 | 20.752 | <0.0001 | *** |
| Cryoprotectant:Antibiotics | 8 | 0.058 | 1.128 | 0.3451 |  |
| Diatom:Cryoprotectant:Antibiotics | 31 | 0.436 | 2.189 | 0.0005 | *** |

## *Models for the individual species:*

Five models (not enough data for *C. meneghiniana*) fitted only interpret p<0.01 as significant. The model per species thus becomes: µ_max_ ~ Cryoprotectant + Antibiotics + Cryoprotectant:Antibiotics.

| *C. closterium* | Df | Sum of Sq | F value | P value | Signif. code |
| --- | --- | --- | --- | --- | --- |
| Cryoprotectant | 8 | 2.608 | 127.21 | <0.0001 | *** |
| Antibiotics | 1 | 0.006 | 2.47 | 0.1223 |  |
| Cryoprotectant:Antibiotics | 8 | 0.051 | 2.5 | 0.0226 | * |

| *O. guenter-grassii* | Df | Sum of Sq | F value | P value | Signif. code |
| --- | --- | --- | --- | --- | --- |
| Cryoprotectant | 8 | 0.0499 | 2.047 | 0.0595 |  |
| Antibiotics | 1 | 0.0778 | 25.526 | <0.0001 | *** |
| Cryoprotectant:Antibiotics | 8 | 0.0452 | 1.854 | 0.0888 |  |

| *P. borealis* | Df | Sum of Sq | F value | P value | Signif. code |
| --- | --- | --- | --- | --- | --- |
| Cryoprotectant | 8 | 0.0642 | 1.46 | 0.1936 |  |
| Antibiotics | 1 | 0.0785 | 14.29 | 0.0004 | *** |
| Cryoprotectant:Antibiotics | 8 | 0.0384 | 0.874 | 0.5441 |  |

| *S. robusta* | Df | Sum of Sq | F value | P value | Signif. code |
| --- | --- | --- | --- | --- | --- |
| Cryoprotectant | 8 | 1.7535 | 20.178 | <0.0001 | *** |
| Antibiotics | 1 | 0.3486 | 32.094 | <0.0001 | *** |
| Cryoprotectant:Antibiotics | 8 | 0.258 | 2.969 | 0.0083 | ** |

| *T. weissflogii* | Df | Sum of Sq | F value | P value | Signif. code |
| --- | --- | --- | --- | --- | --- |
| Cryoprotectant | 7 | 0.2196 | 2.511 | 0.0341 | * |
| Antibiotics | 1 | 0.0369 | 2.957 | 0.0946 |  |
| Cryoprotectant:Antibiotics | 7 | 0.1011 | 1.156 | 0.3532 |  |
